# Medium-term results following arthroscopic reduction in walking-age children with developmental hip dysplasia after failed closed reduction

**DOI:** 10.1186/s13018-017-0635-7

**Published:** 2017-09-21

**Authors:** Liang Zhao, Hua Yan, Changsheng Yang, Daozhang Cai, Yijun Wang

**Affiliations:** Department of Orthopedics, Academy of Orthopedics, Guangdong Province, The Third Affiliated Hospital of Southern Medical University, 183 Zhongshan Avenue West, Guangzhou, 510630 China

**Keywords:** Developmental dysplasia of hip, Unsuccessful closed reduction, Arthroscopic reduction

## Abstract

**Background:**

Arthroscopic reduction has become increasingly popular as an alternative to open reduction for the treatment of developmental dysplasia of the hip (DDH). However, patient outcomes beyond one and a half years after surgery remain unclear. The purpose of this study is to report the medium-term outcomes of walking-age patients who received arthroscopic reduction after an unsuccessful closed reduction. This research was conducted as part of a retrospectively registered study.

**Methods:**

We performed arthroscopic reduction in eight children with DDH after failed closed reduction between January 2010 and January 2012 and followed all cases for a minimum of 5 years. Arthroscopic reduction was performed using a two-portal approach without traction. Capsular release and resection of the transverse acetabular ligament were also performed if needed. Patient demographics, clinical variables, anatomical assessment measures, and post-operative complications were extracted from medical records.

**Results:**

We treated five male and three female patients with an average age at operation of 15.6 months (range, 12 to 22 months). All obstacles to reduction were corrected arthroscopically. Concentric reduction of the hip joint was observed in post-operative X-rays in all cases. The average safe zone was increased from 17.5° (8° to 30°) to 42.1° (36° to 50°) after the operation. The average acetabular (AC) index was reduced from 40.3° (33° to 65°) to 21.9° (19° to 26°) at the end of follow-up. No complications occurred and no patients developed necrosis of the femoral head, recurrent dislocation, or residual hip dysplasia.

**Conclusions:**

Arthroscopic reduction is a suitable surgical procedure for the treatment of DDH among walking-age children with failed closed reduction and severe dislocation. This method is quick and safe, and it can be performed without post-operative complications over the medium term.

## Introduction

Developmental dysplasia of the hip (DDH) is a relatively common hip deformity among infants. Early detection and treatment of DDH are critical to avoid the risk of disability [1]. The application of a Pavlik harness or a spica cast during the first week of life can be effective in most cases. Among infants and young children with DDH, the success rate of early Pavlik harness treatment can be as high as 90% [2]. Unfortunately, many patients, especially those in developing countries, miss this early treatment window. Closed reduction at a later stage is associated with a higher failure rate and can lead to hip instability.

Open reduction, sometimes combined with acetabuloplasty and femoral osteotomy, remains the standard treatment after failed closed reduction [3–6]. However, serious complications [7, 8], including avascular necrosis of the femoral head, may occur following open reduction and negatively affect patient outcomes. Previous studies [9–11] have reported that the rate of necrosis can be as high as 69% if a medial approach is used and up to 30% if an anterior approach is used.

In the search for a less invasive alternative to open reduction, arthroscopic reduction has been performed in several studies [12–15] to treat children with DDH. For example, McCarthy and MacEwen [13] reported the outcomes of three patients with hip dysplasia who received arthroscopic reduction 9 months after the procedure. One patient developed residual dysplasia that required surgery. Eberhardt et al. [14] performed arthroscopic reduction on five very young infants and reported outcomes at a mean follow-up of 13.2 months. A later study by Eberhardt et al. [15] reported the experiences of nine walking-age children who received arthroscopic reduction and acetabuloplasty to treat dislocated hips with a mean follow-up of 15.4 months. However, patient outcomes after a longer time period remain unclear. To fill this research gap, our study assessed the medium-term outcomes of walking-age patients who underwent arthroscopic reduction after an unsuccessful closed reduction.

## Materials and methods

### Study participants

This was a prospective single-centre observational study. The study participants included eight children with DDH after failed closed reduction scheduled to undergo arthroscopic reduction between January 2010 and January 2012 at the Third Affiliated Hospital of Southern Medical University. Surgery indications included patients who underwent a failed closed reduction aged 12 to 24 months, with a magnetic resonance imaging (MRI) scan indicating the presence of intra-acetabular soft tissue or an inverted labrum. The study excluded children over 24 months of age and cases with hip infection (synovial fluid puncture), a comorbid condition (e.g., disease of immune system), or a history of hip surgery (such as acetabuloplasty).

### Medical procedure

All patients were recruited to participate in the study and received an arthroscopic reduction for the treatment of dislocated hips. All procedures were performed by the same surgeon who has extensive experience in adult hip arthroscopic surgery. The procedure was performed under general anesthesia and in a supine position. Arthrography was conducted before the operation to assess the position of the femoral head in relation to other anatomical structures. Two portals without traction were used in all cases. A small pad was placed under the affected hemipelvis. Anatomical landmarks including the femoral artery, the femoral head, the anterior superior iliac spine, and the pubic symphysis were marked prior to incision. With the affected hip in a 90° flexed and 40°–60° abducted position, three Kirschner wires were positioned in parallel spaced 0.5 cm apart and directed inward and downward to the pubic symphysis. The wires were placed above, at the same level of, and below the femoral head. Fluoroscopy was used to guide the initial portal placement. To reduce the arthroscopic puncture using a trocar and reduce the X-ray radiation effects in children, three Kirschner wires were used because we only performed intraoperative fluoroscopy once, when we established an anterolateral portal. One of the three Kirschner wires can be used to position and direct the anterolateral portal’s puncture. We marked direction; the trocar used for the arthroscopic puncture is able to accurately enter the hip.

After an anterolateral portal was marked, a spinal needle was inserted into the hip joint following the previously marked direction. After passing through the tough joint capsule, 20 ml saline was injected. If the needle was successfully placed in the hip joint, the saline fluid was ejected from the needle after removing the syringe. Fluoroscopy was conducted to determine the depth of the spinal needle in the joint cavity. A mark was made on the arthroscope cannula to prevent articular cartilage damage caused by an excessively deep puncture in the joint cavity. A vertical incision of 1 cm was made in the skin with hemostatic forceps, which were used for subcutaneous blunt dissection. An arthroscope puncture trocar was introduced into the hip joint to the depth as marked, and a characteristic “pop” could be felt when penetrating the joint cavity. The arthroscopic sheath was inserted into the joint capsule along the sheath core. In addition, an anterior portal was created where the perpendicular line of the anterior superior iliac spine and the horizontal line of the pubic symphysis met.

**Fig. 1:**
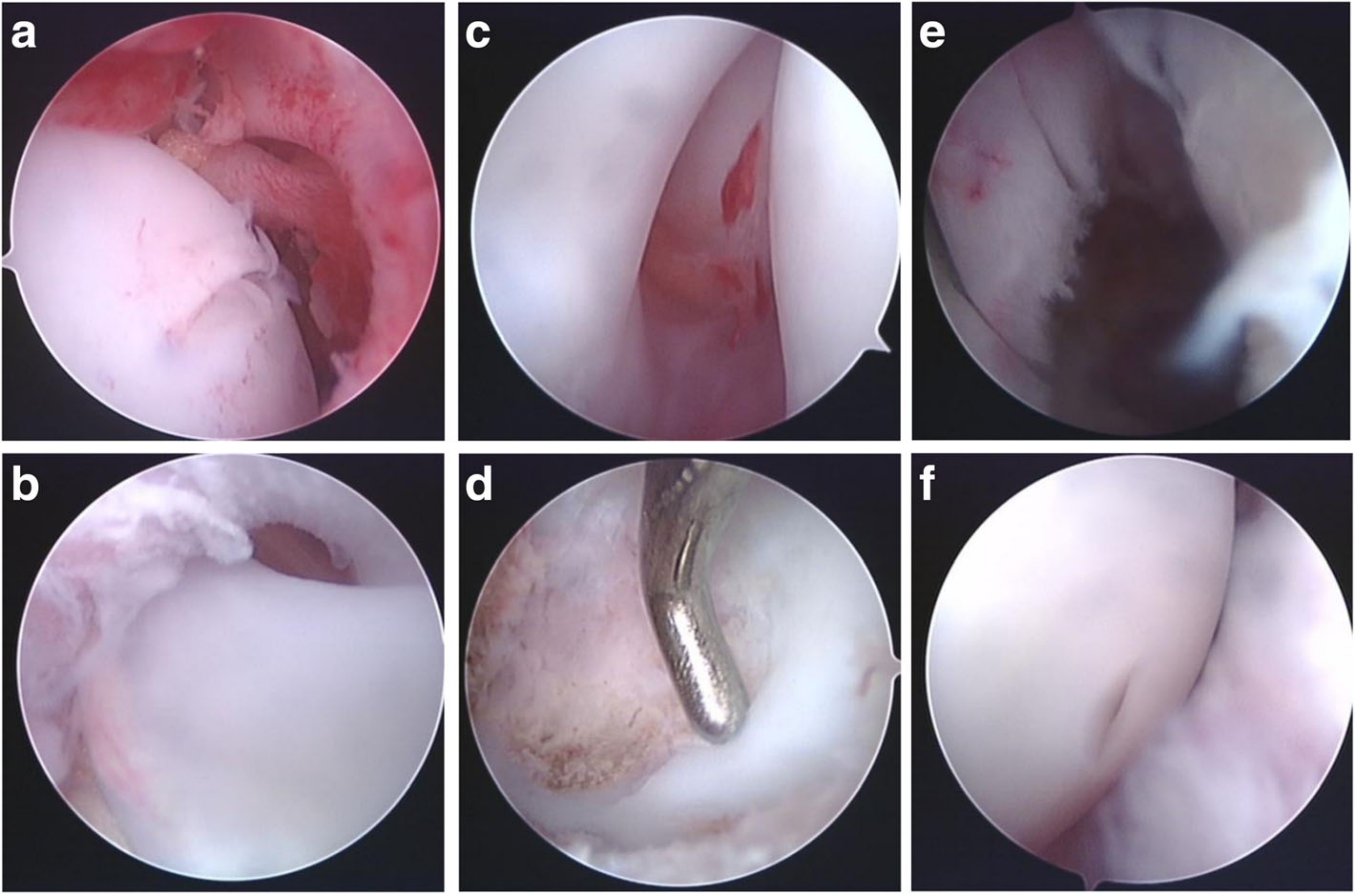
Arthroscopic images of the hip joint. **a** A hypertrophic ligamentum teres. **b** Arthroscopic images of the acetabular fossa and pulvinar tissues. **c** Arthroscopic images of a hypertrophic acetabular labrum. **d** Resection of the acetabular fossa and pulvinar tissue using a shaver. **e** Resection of a hypertrophic acetabular labrum with an electrocautery probe. **f** Proper positioning of the femoral head and the acetabulum following arthroscopic reduction

Arthroscopy was performed using a 4.0-mm, 30° arthroscope. After introduction into the joint cavity, the arthroscope was turned laterally and then in the medial direction to examine the acetabular rim, ligamentum teres, and femoral head. An exploration was conducted to identify obstacles to reduction, including a hypertrophic ligamentum teres (Fig. 1a), fibrofatty or pulvinar tissues (Fig. 1b), and a hypertrophic acetabular labrum (Fig. 1c). The acetabular pulvinar tissue was removed using a shaver (Fig. 1d), and the hypertrophic acetabular labrum and the ligamentum teres was resected with an electrocautery probe (Fig. 1e). After these steps, the horseshoe-shaped articular surface and the acetabular fossa became visible. If a capsular constriction was present, a capsular release was performed with an electrocautery probe. Resection of the transverse acetabular ligament was performed if needed. The surgery lasted 50 ± 10 min in all cases. Proper positioning of the femoral head and the acetabulum were confirmed by X-ray following the arthroscopic reduction procedure (Fig. 1f). A spica cast was applied to retain the hip in a moderately flexed and abducted position for 12 weeks followed by the application of a Pavlik harness to maintain the reduction fixation for 3 to 6 months. During the first year of follow-up, doctor appointments and X-ray examinations were arranged every month. During the second year and thereafter, doctor appointments and X-ray examinations were conducted once per year.

The patient’s gender, age at operation, affected side, previous treatments, and pre-operative Tönnis grade of dislocation were obtained. Anatomical measurements including the safe zone and the acetabular (AC) index were collected both before and after the operation. The AC index refers to the angle formed by Hilgenreiner’s line and a line that extends along the acetabular roofs. A normal AC index is less than 30°. A safe zone was used to assess the stability of the hip joint after arthroscopic reduction, which is defined as the range between the maximum hip abduction angle and the maximum hip adduction angle without dislocation. Safe zone determination was carried out upon hip flexion at 90° after hip joint reduction, followed by recording the full hip abduction angle and the thigh adduction angle to hip joint extrusion. A larger safe zone indicates better stability of the hip joint and vice versa. The ideal safe zone ranges from 30° to 65° [16]. X-rays were obtained at different time points during the follow-up in all cases to monitor the reduction of the hip joint. The post-operative complications [17] included in the analysis were residual hip dysplasia, subluxation or repeated dislocation of the hip, and avascular necrosis of the femoral head.

### Statistical analysis

Data were collected in a Microsoft Excel workbook. Descriptive statistics such as the mean, standard deviation, count, and percent are reported. Paired two-sample Student’s *t* tests were performed using SPSS 22.0 software (IBM, Armonk, NY, USA) to test the statistical significance of the changes in anatomical assessments before and after the operation.

## Results

The study included five male and three female patients, with an average age at operation of 15.6 months (12 to 22 months). All patients were affected unilaterally with five affected hips on the right side and three on the left side. Before arthroscopic reduction, seven patients were treated unsuccessfully with open adductor tenotomy and a spica cast and one failed closed reduction through the application of a Pavlik harness, and all cases were performed serial radiographic studies to monitor whether the reduction is concentric or eccentric for 3 months at least. According to the Tönnis grade of dislocation, two grade III hips and six grade IV hips were included. The main obstacles to reduction included pulvinar tissue, a hypertrophic ligamentum teres, a hypertrophic transverse acetabular ligament, and capsular constriction, which were observed in all eight cases. An inverted labrum, which represents changes to the labrum cartilage complex but is not an obstacle to reduction, was observed in two hips. A pressure lesion in the cartilaginous acetabular roof was observed in all hips, and a neolimbus formation was observed in two hips. Patients’ demographics and pre-operative characteristics are shown in Table 1.

**Table 1:** Patient demographics and pre-operative characteristics

Patient no.	Sex	Age at operation (months)	Affected side	Previous treatment	Tönnis grade of dislocation	Arthroscopic findings						
						Pulvinar tissue	Hypertrophic ligamentum teres	Hypertrophic transverse acetabular ligament	Capsule constriction	Inverted labrum	Pressure lesion in the cartilaginous acetabular roof	Neolimbus
1	M	13	L	Pavlik Harness	III	Yes	Yes	Yes	Yes	Yes	Yes	Yes
2	M	19	R	oAT and spica	IV	Yes	Yes	Yes	Yes	Yes	Yes	Yes
3	M	12	L	oAT and spica	III	Yes	Yes	Yes	Yes	No	Yes	No
4	F	12	L	oAT and spica	IV	Yes	Yes	Yes	Yes	No	Yes	No
5	F	21	R	oAT and spica	IV	Yes	Yes	Yes	Yes	No	Yes	No
6	M	17	R	oAT and spica	IV	Yes	Yes	Yes	Yes	No	Yes	No
7	M	12	L	oAT and spica	IV	Yes	Yes	Yes	Yes	No	Yes	No
8	F	13	L	oAT and spica	IV	Yes	Yes	Yes	Yes	No	Yes	No

*F* female, *M* male, *L* left, *R* right, *oAT* open adductor tenotomy

**Fig. 2:**
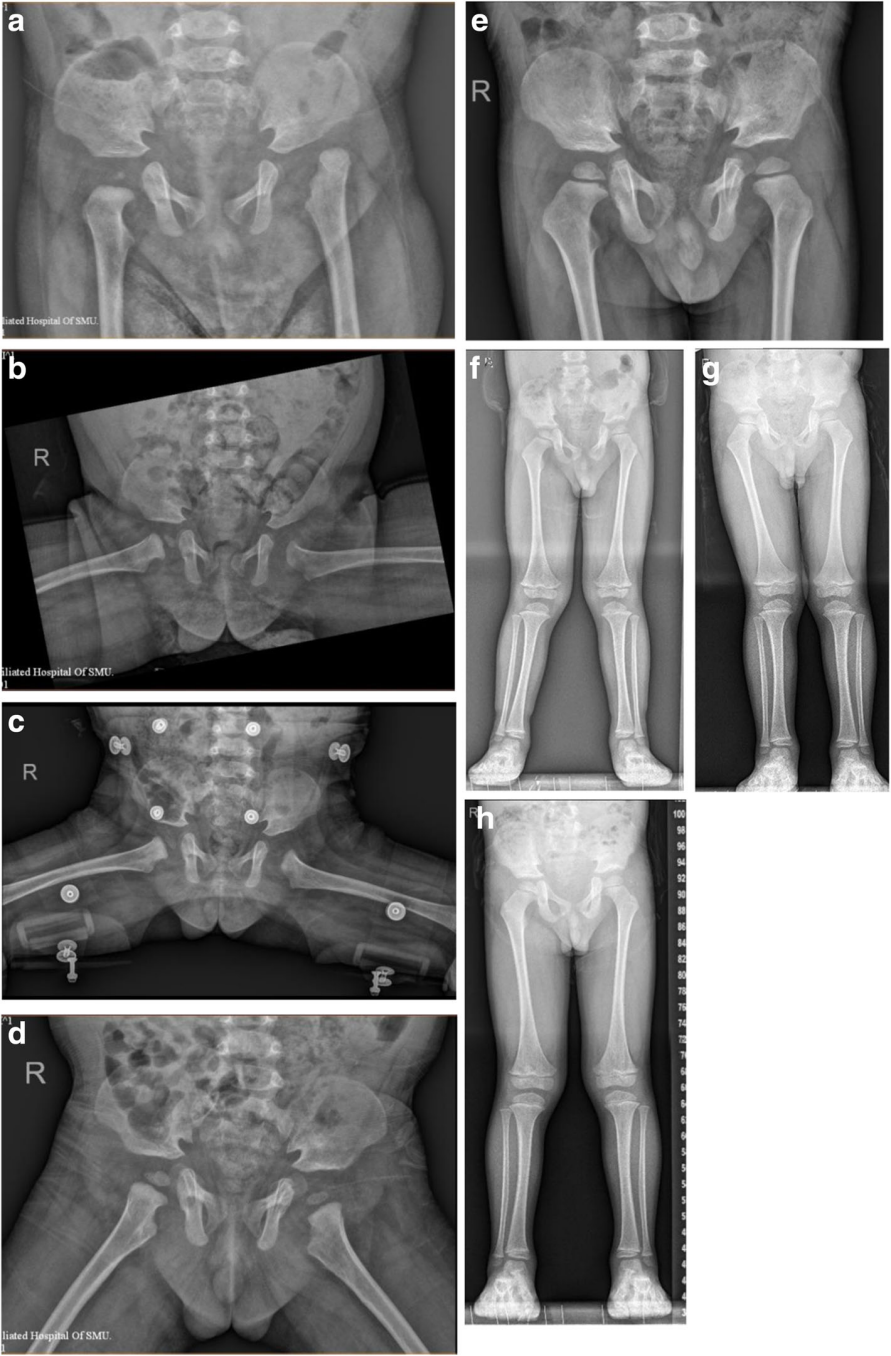
X-ray images of one patient before the operation and during follow-up. **a** Pre-operational imaging. **b** Arthroscopic reduction (concentric reduction) at 1 day after the operation, **c** 3 months of follow-up, **d** 12 months of follow-up, **e** 2 years of follow-up, **f** 3 years of follow-up, **g** 4 years of follow-up, and **h** 5 years of follow-up

Arthroscopic reduction, including the resection of the pulvinar tissue and ligamentum teres, transverse ligament incision, and capsule release, was performed unilaterally in all patients. All reduction obstacles could be arthroscopically eliminated. Concentric reduction of the hip joint was observed on post-operative X-rays in all cases. The average pre-operative safe zone was 17.5° (8° to 30°), while the post-operative safe zone was 42.1° (36° to 50°). Therefore, the safe zone increased by 24.6° on average (95% CI, −30.7, −18.5, *p* < 0.001) after the surgery. The patients were followed-up for a period of 60 months. The average pre-operative AC index was 40.3° (33° to 65°). The average AC index at the final follow-up was 21.9° (19° to 26°), which represents an average decrease of 18.4° (95% CI, 10.4, 26.4, *p* < 0.001). No complications, such as wound hematoma, infection, and neurological or vascular injuries, occurred after surgery. During the follow-up period, none of the patients developed necrosis of the femoral head, and periodic X-ray examinations showed continuous growth of the ossific nucleus of the femoral head. None of the patients developed a recurrent dislocation or residual hip dysplasia. X-ray images of one case before the operation (Fig. 2a) and during follow-up (Fig. 2b–h) are shown. The surgery results and complications during follow-up for individual patients are presented in Table 2.

**Table 2:** Surgery results and complications during follow-up

Patient no.	Concentric reduction on post-op X-ray	Pre-op safe zone (°)	Post-op safe zone (°)	Follow-up period (months)	Pre-op AC Index (°)	AC Index at most recent follow-up (°)	Post-op complications		
							Necrosis of the femoral head	Repeated dislocation of hip	Residual hip dysplasia
1	Yes	18	43	48	35	19	No	No	No
2	Yes	20	40	48	37	21	No	No	No
3	Yes	17	45	48	33	24	No	No	No
4	Yes	18	38	48	38	26	No	No	No
5	Yes	19	40	48	38	23	No	No	No
6	Yes	8	50	48	65	23	No	No	No
7	Yes	10	45	42	36	20	No	No	No
8	Yes	30	36	42	40	19	No	No	No

*AC Index* acetabular index, *Pre-op* pre-operative, *Post-op* post-operative

## Discussion

The treatment for dislocated hips depends on the age of the patient, the degree of dislocation, the anatomical configuration of the proximal femur, and the existing acetabular dysplasia. If the dislocated hip cannot be treated through closed reduction during the first year of life, more extensive treatment is necessary [18]. Although the open reduction remains the standard treatment after failed closed reduction, arthroscopic reduction has been performed in several studies. Despite the increasing number of reports of arthroscopically assisted reduction [13, 19], medium-term outcomes have yet to be reported. Our research findings suggest the medium-term effectiveness of arthroscopic reduction for walking-age children with DDH who failed closed reduction. Further follow-up is warranted to assess the long-term results, as the patients had not reached skeletal maturity.

Seven of eight of our patients previously underwent open adductor tenotomy and spica cast treatment, which failed to restore the femoral head-acetabulum concentricity. Evidence [20] indicates that the failure rate of this treatment can be up to 50%. The high failure rate of closed reduction can be largely attributed to excessive intra-acetabular contents such as hypertrophic fibrofatty (pulvinar) tissue, a thickened ligamentum teres, and an inverted labrum. A small amount of the content may gradually disappear after closed reduction and lead to the formation of femoral head-acetabulum concentricity, allowing the hip joint to return to its normal morphology. In contrast, a large amount of intra-acetabular content will obstruct the repositioning of the femoral head into the acetabulum, which is a major cause of closed reduction failure [21]. One case in our sample failed Pavlik harness treatment. If radiographic assessments show that the hip is not responding to treatment within 3 weeks of application of the harness, the treatment should be discontinued [18].

Various surgical approaches [22–24] have been developed for hip arthroscopy. All arthroscopic reduction procedures in this study were conducted using a two-portal approach with an anterolateral portal and an anterior portal under Kirschner wire-assisted positioning of the hip joint. A similar two-portal approach has been adopted by previous studies [14, 15] to examine the anatomical structure within the hip joint and to perform arthroscopic reduction. To ensure proper positioning and portal placement and to avoid unnecessary soft tissue damage, we used a mobile C-arm machine and multiple Kirschner wires to establish the hip surgical approaches in all cases. We were able to examine all key anatomical structures and remove all obstacles to reduction through the established portals.

The main obstacles to reduction observed in this study included pulvinar tissue, a hypertrophic ligamentum teres, hypertrophic transverse acetabular ligaments, and capsule constriction. These findings are consistent with previous reports [14, 15]. We also observed pressure lesions in the cartilaginous acetabular roof in all cases and an inverted labrum and neolimbus in two cases. These rates are comparable to previously reported rates by Eberhardt et al. [15] who studied older pediatric patients with less severe dislocations.

Bulut et al. [19] reported a combination of arthroscopic reduction and open psoas tenotomy. Unlike Bulut et al., who performed an arthroscopically assisted procedure, we performed a purely arthroscopic reduction. We used arthrography to determine whether the reduction was concentric. All patients reported good outcomes. If the post-arthroscopic reduction was not concentric, psoas tenotomy was performed. All obstacles to reduction were examined and eliminated arthroscopically. In all cases in this study, the hips could be repositioned and stably retained in a Pavlik harness and a spica cast without the use of psoas tenotomy.

Although the medium-term results are encouraging and demonstrate the feasibility of arthroscopic reduction in treating walking-age children with DDH, some limitations of this study should be noted. First, our sample size was relatively small. Second, our study did not have a control group to compare the complication rates associated with other treatment options. Third, this case series reported a single surgeon’s experience, and all cases included in this study were treated at one community hospital. Finally, the age of the included patients ranged from 1 to 2 years. Despite the limitations inherent to this case series, we present the medium-term results of arthroscopic reduction among walking-age children with severe hip dislocation. The results of this study are promising because no arthroscopic-associated complications occurred within an average follow-up period of 5 years. This study, together with other published studies [13–15], demonstrates that arthroscopic reduction is suitable for treating DDH among walking-age children, even those with severe dislocation. Further research that directly compares the results and complications of open reduction to arthroscopic reduction with longer follow-up periods and without psoas tenotomy will be necessary to confirm the efficacy of arthroscopic reduction.

## Conclusions

Arthroscopic reduction is a suitable surgical procedure for the treatment of DDH among walking-age children with failed closed reduction and severe dislocation. It is quicker, safer, and can be achieved without post-operative complications over the medium term.

## Abbreviations

AC index: Acetabular index
DDH: Developmental dysplasia of the hip
MRI: Magnetic resonance imaging
oAT: Open adductor tenotomy
Post-op: Post-operative
Pre-op: Pre-operative
